# Expression and Clinical Significance of the Long Non‐Coding RNA OIP5‐AS1 in Alcohol Use Disorder

**DOI:** 10.1111/adb.70146

**Published:** 2026-03-25

**Authors:** Meng ya Zhu, Jian Wang, Jia Jia Song, Yue Wang, Bo Zhang

**Affiliations:** ^1^ Huai'an No. 3 People's Hospital Huaian Second Clinical College of Xuzhou Medical University Huaian China; ^2^ Department of Psychology Shandong Provincial Hospital Affiliated to Shandong First Medical University Jinan Shandong China

**Keywords:** alcohol use disorder, long non‐coding RNA, OIP5‐AS1

## Abstract

The present study aimed to investigate the expression of OIP5‐AS1 in alcohol use disorder and to explore its potential clinical relevance. A total of 78 patients with alcohol use disorder and 36 healthy controls were enrolled in this study. Clinical data were collected for all participants. The expression levels of OIP5‐AS1 were quantified using quantitative real‐time polymerase chain reaction (RT‐PCR). Receiver operating characteristic (ROC) analysis was subsequently performed to evaluate the diagnostic value of OIP5‐AS1. StarBase‐based bioinformatics analysis suggested that OIP5‐AS1 may function within a miRNA‐mediated regulatory network influencing SERPINA3 expression. OIP5‐AS1 expression levels, as determined by RT‐PCR, were markedly higher in patients with alcohol use disorder than in healthy controls (*p* < 0.001). Patients were stratified into high‐ and low‐expression groups based on the median OIP5‐AS1 level. Comparative analyses of baseline characteristics and clinical parameters showed that body mass index (BMI) was significantly lower in the high‐expression group, and this inverse association between OIP5‐AS1 expression and BMI remained statistically significant in subsequent logistic regression analyses. ROC analysis demonstrated that OIP5‐AS1 had strong diagnostic performance, yielding an area under the curve of 0.9091 (*p* < 0.0001), with a sensitivity of 100% and a specificity of 75% at the defined cutoff. In conclusion, OIP5‐AS1 expression was significantly increased in patients with alcohol use disorder and was inversely associated with BMI. In addition, OIP5‐AS1 demonstrated good diagnostic performance in distinguishing patients with alcohol use disorder from healthy controls. These findings suggest that OIP5‐AS1 may have potential clinical relevance in alcohol use disorder and merit further investigation.

## Graphical abstract

Plasma OIP5‐AS1 is significantly elevated in alcohol use disorder. Higher OIP5‐AS1 expression is associated with lower body mass index and shows good diagnostic performance for distinguishing alcohol use disorder from healthy controls (AUC = 0.9091, sensitivity = 100%, specificity = 75%). These findings support OIP5‐AS1 as a candidate biomarker for alcohol use disorder.

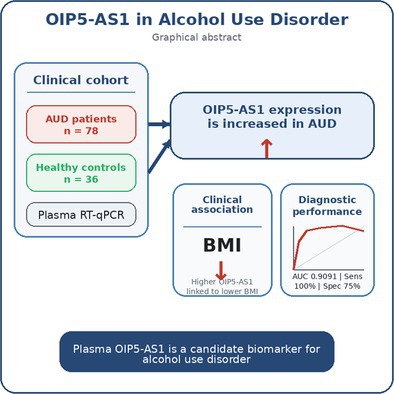

## 1 Introduction

Alcohol use disorder (AUD) is a chronic and relapsing condition characterised by compulsive alcohol consumption and impaired control over alcohol use [1]. AUD poses a significant global health burden and is influenced by both genetic and environmental factors. Twin and adoption studies have consistently estimated the heritability of AUD to be approximately 50%, highlighting a substantial genetic contribution to disease susceptibility [2]. In addition to inherited genetic variation, growing evidence suggests that epigenetic mechanisms play a critical role in mediating the effects of environmental exposures on AUD‐related gene expression. Alcohol‐induced epigenetic alterations, including DNA methylation, histone acetylation and non‐coding RNA regulation, have been implicated in molecular and neurobiological changes associated with AUD and may represent potential biomarkers or therapeutic targets [3].

Long non‐coding RNAs (lncRNAs) are a class of RNA transcripts longer than 200 nucleotides that lack protein‐coding potential [4]. LncRNAs regulate gene expression at transcriptional, post‐transcriptional and epigenetic levels through interactions with DNA, transcription factors, RNAs and chromatin‐associated complexes [5]. Notably, lncRNAs exhibit higher tissue specificity than protein‐coding genes and are particularly enriched in the brain and central nervous system (CNS), suggesting important roles in neural development and function [6]. As key components of gene regulatory networks, lncRNAs have increasingly been implicated in the initiation and progression of AUD [7]. In studies of substance use disorders, lncRNAs—alongside microRNAs—have emerged as major non‐coding RNA species of interest, with accumulating evidence supporting their mechanistic involvement and therapeutic potential in addictive behaviours, including alcohol dependence [8]. Increasing evidence indicates that lncRNAs can function as competing endogenous RNAs (ceRNAs) by acting as molecular sponges for microRNAs, thereby attenuating microRNA‐mediated repression of target mRNA translation [9]. Through this mechanism, lncRNAs participate in complex post‐transcriptional regulatory networks that are sensitive to environmental stimuli. In the context of alcohol exposure, alterations in microRNA and lncRNA expression have been reported to occur via inflammatory signalling pathways such as IL‐6/STAT3, linking non‐coding RNA regulation to neuroinflammatory processes relevant to AUD pathophysiology [10]. OIP5‐AS1 (also known as Oip5os1, 1700020I14Rik or Cyrano) is a pleiotropic lncRNA with broad biological functions across both physiological and pathological contexts, including neurological disorders, cancer and inflammatory diseases. Given its regulatory potential and involvement in inflammation‐ and CNS‐related processes, OIP5‐AS1 may hold translational relevance as a diagnostic, prognostic or therapeutic target in AUD [11].

**FIGURE 1:**
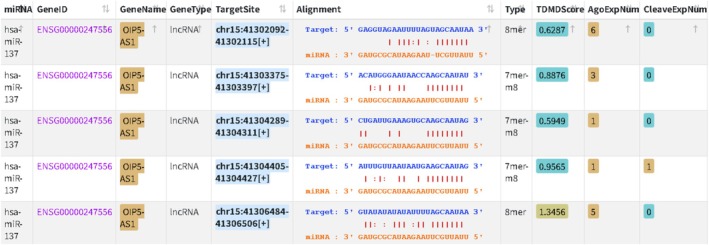
miRNA–lncRNA interactions were predicted using the StarBase database (http://starbase.sysu.edu.cn/), which ultimately identified OIP5‐AS1 as an upstream long non‐coding RNA.

In our previous study, differentially expressed genes associated with AUD were screened using the Gene Expression Omnibus database, leading to the identification of SERPINA3 as a significantly upregulated gene [12]. Subsequent bioinformatics analyses suggested that OIP5‐AS1 (Figure 1) may function as a ceRNA by competitively binding to miR‐137‐3p, thereby attenuating the regulatory effect of miR‐137‐3p on SERPINA3. This interaction may result in increased SERPINA3 expression and promote neuroinflammatory responses. However, the expression profile and potential role of OIP5‐AS1 in AUD remain unclear. Therefore, the present study aimed to investigate the expression and functional relevance of OIP5‐AS1 in AUD.

## 2 Methods

### 2.1 Clinical Samples

Seventy‐eight patients with AUD and 36 age‐ and sex‐matched healthy controls were recruited from the Third People's Hospital of Huai'an City. AUD was diagnosed according to the *Diagnostic and Statistical Manual of Mental Disorders, Fifth Edition*. All participants were aged 18–65 years and underwent semi‐structured clinical interviews conducted by trained psychiatrists. Patients with other substance use disorders (except nicotine dependence), major psychiatric or neurological disorders or severe medical conditions were excluded. All patients were hospitalised for detoxification and had abstained from alcohol for at least 24 h prior to blood sampling. Healthy controls had no history of psychiatric or substance use disorders. Written informed consent was obtained from all participants, and the study was approved by the Ethics Committee of the Third People's Hospital of Huai'an City.

### 2.2 Sample Collection and Processing

Blood samples were collected from fasting participants between 7:00 and 9:00 a.m. Plasma was separated by centrifugation at 3000 × g for 15 min at room temperature. All plasma samples were processed within 30 min of blood collection and stored immediately at −80°C. Each sample underwent only a single freeze–thaw cycle prior to RNA extraction.

### 2.3 Quantitative Real‐Time Polymerase Chain Reaction

Total RNA was isolated from plasma samples of patients with AUD and healthy controls using an RNA extraction kit (Tiangen Biotech, China). RNA was reverse‐transcribed into complementary DNA according to the manufacturer's instructions using a reverse transcription kit (SEVEN, China). Quantitative polymerase chain reaction (PCR) amplification was performed using a standard SYBR Green PCR kit (SEVEN, China). The U6 gene was used as an internal reference. The primer sequences used in this study were as follows: OIP5‐AS1, 5′‐AGGCAGCTTGCTTCTTAATCT‐3′ (forward) and 5′‐ATTCTCAGCCCATGCTGCTT‐3′ (reverse) and U6, 5′‐GCTTCGGCAGCACATATACTAAAAT‐3′ (forward) and 5′‐CGCTTCACGAATTTGCGTGTCAT‐3′ (reverse).

## 3 Statistics

For continuous variables with homogeneity of variance, independent‐samples *t* tests were used to compare differences in laboratory indicators between groups, whereas categorical variables were compared using *χ* ^2^ tests. Logistic regression analysis was further performed to identify variables independently associated with OIP5‐AS1 levels. In the adjusted logistic regression model, body mass index (BMI) was entered as the primary independent variable, with age, sex, marital status and occupation included as covariates to control for potential confounding effects. In addition, a BMI × sex interaction term was tested in a separate model to explore whether sex moderated the association between BMI and OIP5‐AS1 expression.

The diagnostic value of OIP5‐AS1 for AUD was evaluated using receiver operating characteristic (ROC) curve analysis, and the area under the curve (AUC) was calculated. The optimal cutoff value was determined using the Youden index. Each plasma sample was analysed in triplicate, and the mean value was used for statistical analysis to ensure high reproducibility and assay reliability. All statistical tests were two‐sided, and a *p* value < 0.05 was considered statistically significant.

## 4 Results

### 4.1 Experimental Validation of OIP5‐AS1

**FIGURE 2:**
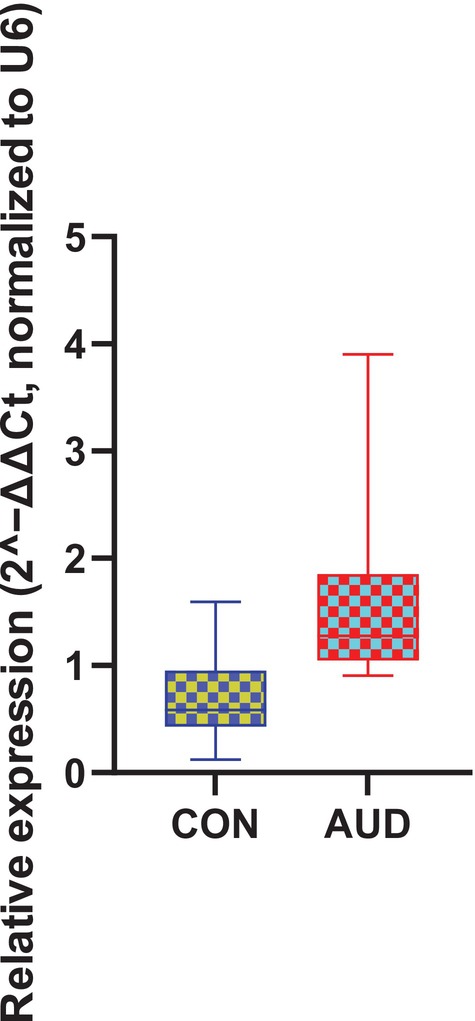
Comparison of plasma OIP5‐AS1 levels between patients with alcohol use disorder and the healthy control group.

Bioinformatics prediction based on the StarBase database identified OIP5‐AS1 as a candidate upstream lncRNA within a miRNA‐mediated regulatory network, which is hypothesised to influence SERPINA3 expression (Figure 1). Quantitative real‐time PCR (RT‐qPCR) analysis demonstrated that OIP5‐AS1 expression levels were significantly higher in patients with AUD than in healthy controls (*p* < 0.001) (Figure 2). The general demographic and clinical characteristics of patients with AUD and healthy controls are summarised in Table 1. Based on the median OIP5‐AS1 expression level, patients were stratified into a high‐expression group (≥ 1.2755, *n* = 39) and a low‐expression group (< 1.2755, *n* = 39). Comparisons of clinical characteristics between the two groups are presented in Table 2. The results showed that BMI was significantly lower in the high OIP5‐AS1 expression group, indicating an inverse association between OIP5‐AS1 expression and BMI (*p* = 0.005). Further binary logistic regression analysis confirmed that the difference in BMI between the two groups remained statistically significant (*p* = 0.009) (Table 3). To further examine whether sex moderated the association between BMI and OIP5‐AS1 expression, an additional regression model including BMI, sex and their interaction term (BMI × sex) was fitted. The interaction term was not statistically significant (*β* = −0.001, *p* = 0.513), and the model explained a limited proportion of variance (adjusted *R* ^2^ = 0.056), indicating no evidence of a sex‐specific moderation effect in the present sample.

**TABLE 1:** Comparison of general characteristics between the alcohol use disorder (AUD) group and the healthy control group.

	Alcohol use disorder (*n* = 78)	Health control (*n* = 36)	*p*
Demographics			
Age, years	49 ± 10	46 ± 12	0.144
Gender			0.890
Male, *n* (%)	72 (92.3)	34 (94.4)	
Female, *n* (%)	6 (7.7)	2 (5.6)	
BMI, kg/m^2^	21.2 ± 4.2	22.4 ± 2.8	0.127

**TABLE 2:** Comparison of demographic and clinical characteristics between the high and low OIP5‐AS1 expression groups.

	High OIP5‐AS1 level (*n* = 39)	Low OIP5‐AS1 level (*n* = 39)	*p*
Demographics			
Age, years	46 ± 10	47 ± 11	0.148
Gender			0.089
Male, *n* (%)	34 (87.2)	38 (97.4)	
Female, *n* (%)	5 (12.8)	1 (2.6)	
Education (years), *n* (%)			0.917
≤ 6	26 (68.4)	25 (67.6)	
6–12	7 (18.4)	6 (16.2)	
≥ 12	5 (13.2)	6 (16.2)	
Marital status, *n* (%)			0.399
Unmarried	3 (7.7)	3 (7.7)	
Married	30 (76.9)	26 (66.7)	
Divorced	5 (12.8)	10 (25.6)	
Widowed, *n* (%)	1 (2.6)	0 (0.0)	
Occupation, *n* (%)			0.319
Unemployed	33 (84.6)	30 (76.9)	
Physical labour	4 (10.3)	3 (7.7)	
Mental labour	2 (5.1)	6 (15.4)	
BMI, kg/m^2^	19.7 ± 3.00	22.5 ± 4.77	0.005*
Drinking duration (years)	21 ± 10	23 ± 10	0.404
Heart rate, bpm	97 ± 14	95 ± 16	0.667
Systolic blood pressure, mmHg	138 ± 20	136 ± 18	0.709
Diastolic blood pressure, mmHg	90 + 14	87 ± 11	0.249
Smoking duration (years)	9 ± 13	14 ± 15	0.175
**Laboratory tests**			
RBC	3.97 ± 0.629	4.17 ± 0.730	0.220
Haemoglobin, g/L	131.08 ± 18.541	135.13 ± 19.802	0.354
MCV	99.03 ± 7.135	98.31 ± 5.642	0.619
WBC count, ×109/L	6.81 ± 2.151	6.59 ± 2.013	0.646
Neutrophils, ×10^9^/L	4.36 ± 2.019	4.01 ± 1.862	0.429
Lymphocytes, ×10^9^/L	1.74 ± 0.672	1.88 ± 0.728	0.410
Platelets, ×10^9^/L	205.44 ± 83.332	225.67 ± 103.018	0.343
AST, U/L	58.9 ± 60.024	65.79 ± 73.942	0.655
ALT, U/L	49.55 ± 61.194	46.37 ± 47.503	0.799
GGT, U/L	213.39 ± 349.104	221.78 ± 391.575	0.921
TBIL, μmol/L	20.89 ± 19.100	20.38 ± 14.301	0.895
DBIL, μmol/L	7.99 ± 12.506	7.37 ± 6.766	0.786
IBIL, μmol/L	12.9 ± 8.416	13.01 ± 8.600	0.954
Serum creatinine, μmol/L	60.53 ± 16.984	57.37 ± 16.257	0.415
UA, μmol/L	352.04 ± 126.890	332.21 ± 145.301	0.513
BUN, mmol/L	4.09 ± 1.467	4.26 ± 1.775	0.645
CK, U/L	726.32 ± 2400.116	678.22 ± 2379.598	0.935
CK‐MB, U/L	22.17 ± 35.202	20.90 ± 32.798	0.879

*Note:* Continuous variables were compared using independent‐samples *t* test or Mann–Whitney *U* test, as appropriate, and categorical variables were compared using *χ* ^2^ tests.

* Statistically significant difference.

**TABLE 3:** Binary logistic regression models for OIP5‐AS1.

	*B*	SE	Sig.	Exp(*B*)	95% CI	
					Lower	Upper
BMI	0.203	0.078	0.009	1.225	1.051	1.429
Constant	−4.201	1.641	001	0.015		

Abbreviations: CI, confidence interval; SE, standard error.

### 4.2 Diagnostic Value of OIP5‐AS1

**FIGURE 3:**
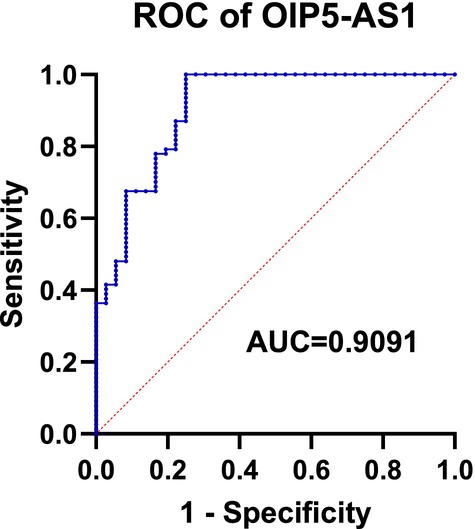
Receiver operating characteristic (ROC) curve based on OIP5‐AS1 levels in patients with alcohol use disorder. The area under the curve (AUC) for OIP5‐AS1 was 0.9091 (*p* < 0.0001), with a sensitivity of 100% and a specificity of 75% at an optimal cutoff value of 0.75, as determined by the Youden index.

ROC analysis was used to test the diagnostic value of OIP5‐AS1, wherein it revealed that OIP5‐AS1 had an AUC of 0.9091 (*p* < 0.0001), sensitivity of 100% and specificity of 75% (Figure 3).

## 5 Discussion

AUD is a multifactorial condition shaped by the interplay between genetic susceptibility and environmentally driven molecular adaptations. Growing evidence highlights the pivotal role of epigenetic regulation—particularly non‐coding RNAs—in the pathophysiology of AUD [13]. In the present study, we examined the expression pattern and potential clinical relevance of the lncRNA OIP5‐AS1 in patients with AUD. Using RT‐qPCR analysis, we demonstrated that OIP5‐AS1 expression was significantly elevated in individuals with AUD compared with healthy controls, providing direct clinical evidence of its dysregulation in AUD and extending previous bioinformatics‐based predictions. Given that lncRNAs are highly enriched in the CNS and exhibit pronounced tissue and cell‐type specificity [14], altered OIP5‐AS1 expression may reflect disease‐associated molecular changes linked to chronic alcohol exposure. Notably, OIP5‐AS1—also known as Cyrano according to NCBI Gene and Ensembl annotations—is an evolutionarily conserved lncRNA with high expression in the CNS [15], further supporting its potential relevance to neurobiological processes underlying AUD.

In neuroscience research, lncRNAs have been shown to play critical roles in neuronal differentiation, maintenance of synaptic function and regulation of neural circuit homeostasis, and their dysregulated expression has been consistently observed across a broad spectrum of neurological diseases and psychiatric disorder models [16]. These findings underscore the importance of lncRNAs in preserving molecular homeostasis within the nervous system. In this context, OIP5‐AS1, as a lncRNA with well‐defined expression characteristics and regulatory potential in neural tissues, may participate in adaptive cellular responses to both intrinsic and extrinsic environmental challenges. Importantly, chronic alcohol exposure is known to induce widespread and long‐lasting transcriptomic and epigenetic remodelling [17], with pronounced effects on neural plasticity [18], stress‐response systems [19] and metabolism‐related pathways [20, 21]. Against this background, the observed upregulation of OIP5‐AS1 is more likely to reflect a disease‐associated reorganisation of molecular networks under sustained alcohol insult. Accordingly, alterations in OIP5‐AS1 expression not only carry potential pathological significance but also provide a biologically plausible rationale for considering OIP5‐AS1 as a candidate molecular biomarker and/or regulatory node in the pathophysiology of AUD.

Emerging evidence indicates that lncRNAs play critical roles in the regulation of systemic metabolism and energy homeostasis through both transcriptional and post‐transcriptional mechanisms [22, 23, 24]. In line with this notion, OIP5‐AS1 has been implicated in multiple biological processes closely linked to metabolic regulation, including mitochondrial function, cellular stress responses and inflammatory signalling pathways [25, 26]. Notably, these processes are profoundly disrupted in the context of AUD, as chronic ethanol exposure is known to induce substantial metabolic derangements, such as altered lipid utilisation [27], insulin resistance [20] and progressive weight loss [28].

In the present study, we observed a significant inverse association between OIP5‐AS1 expression and BMI, with lower BMI values detected in patients exhibiting higher OIP5‐AS1 levels. Importantly, this relationship remained statistically significant after adjustment for potential confounders in logistic regression analyses. Although the precise molecular mechanisms underlying this relationship remain to be elucidated, these findings raise the possibility that OIP5‐AS1 dysregulation may be linked to metabolic or nutritional alterations that are commonly observed in individuals with AUD.

Previous studies have demonstrated that chronic alcohol consumption disrupts metabolic homeostasis and inflammatory signalling—two tightly interconnected processes in which non‐coding RNAs are increasingly recognised as key regulatory elements [29]. Clinically, AUD is frequently accompanied by nutritional insufficiency, altered appetite regulation, impaired nutrient absorption and systemic catabolic states, all of which may contribute to reduced BMI.

It should also be acknowledged that BMI represents a relatively coarse surrogate for nutritional and metabolic status. Consequently, future studies incorporating more granular phenotypic measures—such as body composition analysis, dietary intake assessment, liver function indices, inflammatory biomarkers and metabolic hormones—will be necessary to determine whether OIP5‐AS1 is more closely associated with malnutrition, hepatic dysfunction, systemic inflammation or other metabolic domains relevant to AUD. Such integrative approaches may help clarify whether OIP5‐AS1 functions as an active regulator within alcohol‐related metabolic pathology or reflects a downstream molecular signature of systemic metabolic stress.

In addition, ROC curve analysis demonstrated that OIP5‐AS1 exhibited good diagnostic performance in distinguishing patients with AUD from healthy controls, with a high AUC, sensitivity and specificity. These results indicate that OIP5‐AS1 may have potential value as a biomarker for AUD. Importantly, this diagnostic performance was achieved using peripheral plasma samples, suggesting that OIP5‐AS1 could serve as a minimally invasive indicator of disease status.

Previous studies have suggested that OIP5‐AS1 may function as a ceRNA involved in inflammatory and neurobiological pathways. In our earlier bioinformatics analyses, OIP5‐AS1 was predicted to interact with miR‐137‐3p and modulate the expression of SERPINA3, a gene implicated in inflammatory responses. Although the present study did not directly investigate this regulatory axis, the observed upregulation of OIP5‐AS1 in AUD patients is consistent with a potential role in AUD‐related neuroinflammatory processes and warrants further mechanistic investigation.

Several limitations of this study should be acknowledged. First, the sample size was relatively modest and all participants were recruited from a single centre, which may limit the generalizability of the findings. Second, although a BMI × sex interaction was tested, no significant sex‐moderating effect was observed; sex‐stratified analyses were not performed due to the small number of female participants, and future studies with larger, sex‐balanced samples are warranted. Third, the cross‐sectional design and the lack of systematically collected AUD severity measures constrained causal inference and precluded more refined analyses of the relationship between OIP5‐AS1 expression and clinical features of AUD. Finally, functional validation experiments were not performed, which limits mechanistic interpretation of the observed associations. Accordingly, future studies may incorporate standardised assessments of AUD severity and functional approaches, such as OIP5‐AS1 knockdown or overexpression in relevant cellular models, together with integrative analyses of lncRNA–miRNA–mRNA networks and inflammation‐ or metabolism‐related pathways to further clarify downstream targets and the biological relevance of OIP5‐AS1 in AUD.

In summary, the present findings indicate that OIP5‐AS1 is significantly elevated in AUD, is negatively associated with BMI and exhibits promising diagnostic performance. Collectively, these results support OIP5‐AS1 as a candidate biomarker for AUD and highlight a potentially meaningful link between AUD‐related molecular changes and metabolic status. Further validation and mechanistic studies are warranted to define its clinical utility and biological relevance.

## 6 Conclusion

In conclusion, this study demonstrates that OIP5‐AS1 expression is significantly increased in patients with AUD and is inversely associated with BMI. Moreover, OIP5‐AS1 shows good diagnostic performance in distinguishing patients with AUD from healthy controls. These findings suggest that OIP5‐AS1 may have potential clinical relevance in AUD and provide a basis for further investigation into its biological function and potential utility as a biomarker.

## Author Contributions

Meng ya Zhu and Jian Wang collected the data and conducted the experiments. Jia Jia Song and Yue Wang participated in the statistical analysis and reference collection. Bo Zhang drafted the manuscript and performed revisions.

## Funding

The authors have nothing to report.

## Ethics Statement

All participants provided written informed consent. The study was authorised and approved by the Third People's Hospital Ethics Committee of Huai'an and carried out in compliance with the Declaration of Helsinki (IRB Number: 2021‐07).

## Consent

The authors have nothing to report.

## Conflicts of Interest

The authors declare no conflicts of interest.

## Data Availability

The original contributions presented in the study are included in the article; further inquiries can be directed to the corresponding author.
